# Regulation of gut epithelial barrier and tuft/goblet cell responses by microbiome repair: Opportunities and future directions

**DOI:** 10.1073/pnas.2535289123

**Published:** 2026-06-08

**Authors:** Zian Wang, Jiahao Xie, Shujing Liu, Mei Han, Liang Wang

**Affiliations:** ^a^ Research and Teaching Department of Comparative Medicine, Dalian Medical University, Dalian City, Liaoning Province 116044, China; ^b^ Department of Gastroenterology, The Second Hospital of Dalian Medical University, Dalian City, Liaoning Province 116023, China

Recently, Wang et al. discovered that microbiome-directed complementary food (MDCF-2)–mediated repair of the gut microbiota improved growth outcomes in undernourished children and, in gnotobiotic mice, restored tuft and goblet cell phenotypes, increased cecal butyrate levels, and enhanced mucin glycosylation (1). This work provides important experimental evidence for exploring microbiome interventions aimed at improving intestinal barrier function and immune homeostasis.

**Figure:**
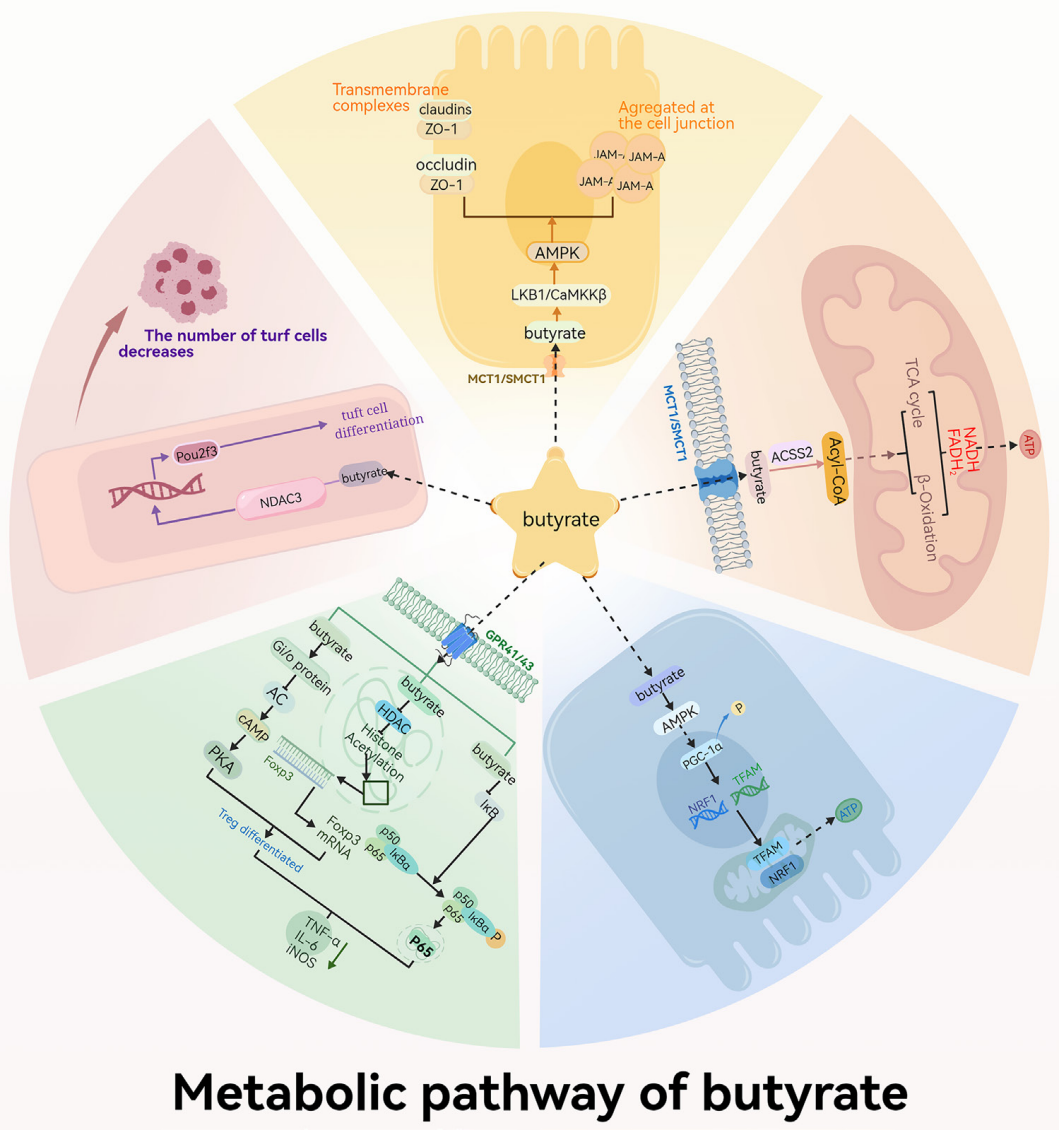
Butyrate exerts its beneficial effects on gut repair through five synergistic pathways: 1) Immunomodulation: Inhibits HDAC3 in intestinal stem cells, downregulates Pou2f3, and reduces tuft cell differentiation and type 2 immune responses; 2) Barrier enhancement: Activates AMPK in enterocytes, promotes membrane assembly of ZO-1 and JAM-A, and decreases intestinal permeability; 3) Energy metabolism: Generates ATP via β-oxidation and the TCA cycle to fuel cellular functions; 4) Anti-inflammatory effects: Suppresses proinflammatory cytokines (TNF-α, IL-6) from immune cells and promotes Treg differentiation through GPR41/43 signaling, HDAC inhibition, and NF-κB blockade; 5) Mitochondrial biogenesis: Activates the AMPK–PGC-1α axis, upregulates NRF1 and TFAM, and enhances long-term energy reserve in intestinal epithelial cells. Created with BioGDP.com (3).

Although metabolites such as butyrate are implicated in modulating tuft and goblet cells, the causal relationships between specific microbial metabolites and distinct epithelial cell types remain to be fully elucidated. For instance, recent evidence suggests that microbiota-derived butyrate can suppress tuft cell differentiation via inhibition of histone deacetylase 3 (HDAC3) in intestinal stem cells, thereby modulating type 2 immunity (2). Future studies employing single-cell metabolomics or metabolite-tracing approaches could more precisely identify key microbial effectors and their target pathways, providing deeper mechanistic insights.

Moreover, the study demonstrates increased expression of tight junction proteins (ZO-1, JAM-A) following microbiome repair; however, the long-term stability and functional consequences of these changes are not yet fully established. Complementary assays assessing gut permeability (e.g., transepithelial resistance), responses to inflammatory challenges, or microbiome withdrawal experiments could further clarify the durability and protective relevance of microbiome-driven barrier enhancement. Notably, prior work shows that butyrate can enhance tight junction assembly via activation of AMP-activated protein kinase (AMPK) in intestinal epithelial cells, promoting redistribution of junctional proteins ZO-1 and occludin to cell peripheries (4), supporting the plausibility of such protective effects.

Butyrate also regulates goblet cell function and mucin production via the wingless-related integration site 1/extracellular signal-regulated kinase signaling pathway (5). In addition to its barrier-protective properties, butyrate promotes mitochondrial biogenesis by inducing the expression of peroxisome proliferator-activated receptor gamma coactivator (PGC)-1α and its downstream effectors, nuclear respiratory factor 1 (NRF1) and transcription factor A, mitochondrial (TFAM). This, in turn, enhances oxidative metabolism and increases respiratory capacity in colonic epithelial cells (6, 7). These metabolic adaptations may underpin the sustained improvements in energy homeostasis and growth observed after microbiota restoration, which persist beyond the period of active butyrate production.

In summary, this work provides compelling evidence supporting the benefits of microbiome-directed interventions in promoting gut barrier integrity and growth. Systematic exploration of developmental stage–specific effects, metabolite causality, barrier function validation, and microbiome complexity will further strengthen the translational potential of such strategies.
